# *Periplaneta americana* Extract Pretreatment Alleviates Oxidative Stress and Inflammation and Increases the Abundance of Gut *Akkermansia muciniphila* in Diquat-Induced Mice

**DOI:** 10.3390/antiox11091806

**Published:** 2022-09-13

**Authors:** Shiyi Lu, Shuyi Xu, Lingjun Chen, Yuhang Deng, Jie Feng

**Affiliations:** Key Laboratory of Animal Nutrition & Feed, College of Animal Sciences, Zhejiang University, Hangzhou 310058, China

**Keywords:** PAE (*Periplaneta americana* extract), diquat, oxidative stress, inflammation, gut microbiota, *Akkermansia muciniphila*, mice

## Abstract

Studies have shown that *Periplaneta americana* extract (PAE) has good therapeutic effects in inflammatory disorders such as ulcerative colitis, alcoholic hepatitis, and gastric ulcers. However, whether or not PAE has good pre-protective effects has not been widely and deeply studied. In this study, we investigated the effects of PAE pretreatment for 7 days on oxidative stress and inflammation triggered by oxidative stress by using diquat-induced C57BL/6 mice as an oxidative stress model. The results showed that PAE pretreatment could significantly reduce oxidative stress in the intestine and liver by reducing the production of MDA, and improved antioxidant systems (SOD, CAT, GSH, and T-AOC). By primarily activating the anti-inflammatory cytokine (IL-10) mediated JAK1/STAT3 signaling pathway, PAE also effectively reduced oxidative stress-induced liver inflammation while also reducing liver damage, as evidenced by the reductions in serum AST and ALT. PAE pretreatment also had a significant effect on maintaining the intestinal barrier function, which was manifested by inhibiting a decrease in the expression of tight junction proteins (ZO-1 and occludin), and reducing the increased intestinal permeability (serum DAO and D-Lac) caused by diquat. The 16S rRNA sequencing analysis revealed that diquat decreased the gut microbiota diversity index and increased the abundance of pathogenic bacteria (e.g., *Allobaculum*, *Providencia* and *Escherichia-Shigella*), while PAE pretreatment responded to diquat-induced damage by greatly increasing the abundance of *Akkermansia muciniphila*. These findings elucidate potential pre-protective mechanisms of PAE in alleviating oxidative stress and inflammation, while providing a direction for the treatment of metabolic diseases by utilizing PAE to enhance the abundance of gut *A. muciniphila*.

## 1. Introduction

Oxidative stress is defined as an imbalance between the production of free radicals and reactive metabolites, also known as reactive oxygen species (ROS) or oxidants, and their elimination by the antioxidative protection systems [1,2]. Antioxidant enzymes resist increases in ROS levels in combination with non-enzymatic antioxidants [3]. The nuclear factor erythroid 2-related factor 2 (Nrf2), a regulator of cellular resistance to oxidants, regulates the physiological and pathological effects of oxidant exposure by controlling the basal and induced expression of an array of antioxidant response element-dependent genes [4]. Extensive studies have shown that continued oxidative stress can lead to chronic inflammation, which can further induce some diseases such as Alzheimer’s, cancer, diabetes, inflammatory diseases, and the aging process [1]. In the pathophysiology of hypertension, oxidative stress and inflammation work in a cooperative and synergistic manner. Inappropriate oxidative stress increases inflammatory responses, augmenting blood pressure and causing damage to cardiovascular control organs [5]. Oxidative stress and inflammation trigger atherothrombosis, which illustrates the activation of immune cells and recruitment to vascular tissues by classical cardiovascular risk factors, leading to the activation of secondary vascular ROS sources [6]. There are also studies that have shown that treating cardiovascular disease with a combination of antioxidant and anti-inflammatory therapy may hold great promise [7]. In addition, obesity is linked to a low-grade proinflammatory state, which may involve impaired antioxidant and oxidative stress pathways [8,9].

The small intestine features the largest mucosal surface within the body. The intestinal epithelium transports solutes and water between lumen and blood, while also forming a highly selective barrier between these compartments to restrict the passage of harmful molecules, bacteria, viruses, and other pathogens. The intestinal barrier, which includes a mucus layer and a monolayer of epithelial cells interconnected by tight junctions (TJs), is essential for maintaining intestinal homeostasis [10], and the functional status of intestinal barrier is described by intestinal permeability [11]. Re-localization and altered expression of TJ protein subunits can cause the rapid deterioration of intestinal barrier function, impairing normal secretory and absorptive functions as well as removing the host’s physical barrier to harmful luminal stimuli [12]. The intestinal microbiota refers to the group of microorganisms living in the animal intestine that establish a symbiotic relationship with their host. The functions of microbiota can be grouped into three main categories: protective, trophic, and metabolic. These functions play roles in maintaining epithelial cell integrity and contribute to maintain the host defense system at homeostasis by producing antimicrobial substances, promoting mucus secretion, modifying pH, and preventing pathogen colonization [13]. The liver, a key frontline immune tissue, is able to mount a rapid and powerful immune response, since it contains the body’s largest collection of phagocytic cells [14]. Inflammation is trigged to facilitate tissue repair in the case of acute liver injury. Likewise, the liver can quickly transition from immunological hyporesponsiveness to produce a potent inflammatory response and effective adaptive immunity when the level or context of microbial products changes [14]. The liver and intestinal system are closely related. The liver’s crucial location is downstream of the gut, where portal venous blood drains; therefore, the liver is frequently exposed to nutrients, poisons, antigens produced from food, microbial byproducts, and intestinal tract bacteria which have been found in people whose liver functions have been seriously compromised [14,15,16]. This suggests that the liver performs crucial functions such as eliminating translocated microorganisms. The liver can act as a functional vascular firewall to be a second barrier limiting and clearing commensals and pathogenic bacteria that penetrate the gut [17]. Numerous studies have now demonstrated that the gut microbiota were associated with many diseases, especially those related to the gastrointestinal tract and liver, such as intestinal bowel disease (IBD), irritable bowel syndrome (IBS), colorectal cancer (CRC), as well as alcoholic liver disease, non-alcoholic liver disease, and primary sclerosing cholangitis [18,19].

*Periplaneta americana* L. is an insect of the genus *Periplaneta* in the family Blattidae, and is a type of characteristic traditional Chinese medicine, whose medicinal use was first recorded in the *Shen Nong’s Materia Medica* of the Eastern Han dynasty (A.D. 25–220) [20]. In the *Compendium of Materia Medica* of the Ming Dynasty (A.D. 1578), there is also a description of the use of *Periplaneta americana* as a drug that can promote blood circulation, detoxification, and urination [20]. The preparations of *Periplaneta americana* are widely used in clinical practice, especially its bioactive extracts. *Periplaneta americana* extract (PAE) is a mixture obtained by extracting *Periplaneta americana* with ethanol, comprising polyols, dopamine, peptides (such as thymosin, dipeptide, and antimicrobial peptides), nucleosides, viscous sugar amino acids, amino acids, coumarin, epidermal growth factors, and other unclear active components [21,22,23]. PAE can promote wound healing and has been explored in some inflammatory diseases with a good therapeutic effect, such as ulcerative colitis (UC), gastric ulcer, alcoholic hepatitis, and chronic hepatitis B [21,23,24,25,26]. At present, the research, generally, has focused on the treatment and repair effects of PAE, while the research on the pre-protective effect has been limited. At the same time, there are few reports on the effect of PAE on the gut microbiome. 

Diquat (1,1′-ethylene-2,2′-bipyridilium), a redox cycling compound, is easily transformed into a free radical, which reacts with molecular oxygen to generate superoxide anion (O_2_^•^^−^) and then other oxygen products, such as hydrogen peroxide (H_2_O_2_) and hydroxyl radical (·OH) [27]. Diquat is now commonly employed to induce oxidative stress in vivo and in vitro. Therefore, the diquat-induced oxidative stress model in C57BL/6 mice was employed in this investigation, in order to explore the pre-protective effects of PAE on oxidative stress, inflammation, and gut microbes.

## 2. Materials and Methods 

### 2.1. Periplaneta Americana Extract

The *Periplaneta americana* extract (PAE) was provided by Zhejiang Jingxin Pharmaceutical Co., Ltd. (Shaoxing, China). The *Periplaneta americana* powder was extracted with 90% ethanol, three times at 60 °C, each time for 24 h. The solution was combined, filtered, and concentrated to be ethanol free. Then, the obtained concentrated solution was added to an equal volume of double-distilled water. After 24 h, the precipitate and oil layer were separated and discarded, the aqueous solution was filtered and concentrated under reduced pressure to a relative density of 1.15–1.20 at 70 °C to obtain PAE (the purity ≥ 93%). The quality identification standard of PAE is to determine the content of alanine (≥7%) by UV-visible spectrophotometry. The content of alanine in this experimental sample was 7.35%.

### 2.2. Animal Experiments

**Figure 1:**
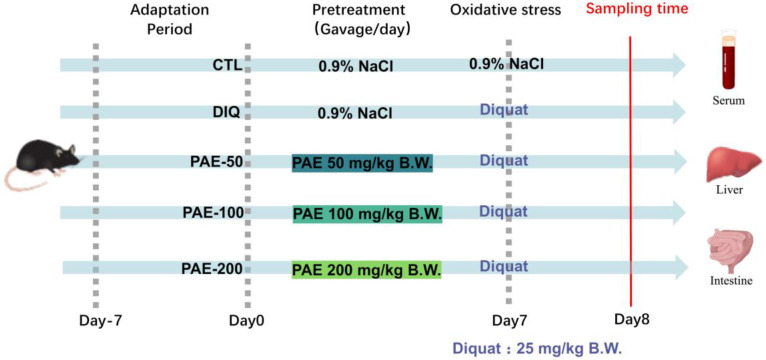
Experimental scheme for assigning mice into 5 groups (*n* = 8).

All animal studies were performed in accordance with the guidelines of the Animal Ethics Committee of Zhejiang University (Ethical protocol code ZJU20160403). Forty C57BL/6 mice (20 ± 2 g), 8-week-old males, were purchased from Slac Laboratory Animal Co., Ltd. (Shanghai, China). As shown in the scheme (Figure 1), after 7 days of adaptation, the mice were randomly divided into 5 groups (*n* = 8 per group): a control group (CTL), the diquat model group (DIQ), the low dose of PAE group (PAE-50), the medium dose of PAE group (PAE-100), and the high dose of PAE group (PAE-200). By oral gavage, the control and diquat groups were given 0.9% NaCl (normal saline), and the PAE-50/100/200 groups were given PAE (50/100/200 mg/kg B.W.), respectively, at 9:00 a.m. for seven days (Days 0–6). On Day 7, the mice in the control group were administrated saline by intraperitoneal injection, meanwhile other groups were injected diquat (25 mg/kg B.W., Dr. Ehrenstorfer GmbH, Augsburg, Germany), and the samples were collected after 24 h. During the experiment, the mice were fed with a basal diet and kept in 12 h light/dark cycle at 20–26 °C.

### 2.3. Sample Collection and Pretreatment Protocol

After the diquat treatments for 24 h, the experimental mice were anesthetized with 4% chloral hydrate, and blood was quickly collected by extracting the eyeballs. The serum samples were separated by centrifugation at 3000 rpm for 10 min at 4 °C, after standing at room temperature for 1 h. Meanwhile, the abdominal cavity was opened, and tissues were quickly collected, frozen in liquid nitrogen, and stored at −80 °C. Subsequent biochemical analysis used serum, jejunum, and liver samples, and 16S rRNA sequencing used cecum samples.

### 2.4. Oxidative Stress Parameter

#### 2.4.1. Determination of GSH and MDA Concentrations

The lysed cell samples were centrifuged at 12,000 rpm for 5 min at 4 °C to obtain the supernatant for testing. Tissue samples were made into a 10% homogenate and centrifuged at 12,000 rpm for 5 min at 4 °C to obtain the supernatant for testing. The concentration of protein was determined by BCA assay (KeyGen, Nanjing, China). Thereafter, the concentrations of glutathione (GSH) and malondialdehyde (MDA) were determined using a GSH assay kit (A006-1-1) and MDA assay kit (A061-2-1) (Jiancheng, Nanjing, China), respectively. 

#### 2.4.2. Determination of SOD, CAT, and T-AOC Activities

The activities of catalase (CAT, A007-1-1) and the total antioxidant capacity (T-AOC, A015-3-1) were determined according to the instructions of the assay kits (Jiancheng, Nanjing, China), and superoxide dismutase (SOD, S0101S) was determined according to the instructions of the assay kits (Beyotime, Nanjing, China). 

### 2.5. Determination of Inflammatory Factor

The levels of tumor necrosis factor alpha (TNF-α, H025-1), interleukin (IL)-1β (H002-1), IL-10 (H009-1), and IL-22 (H019) were determined using enzyme-linked immunosorbent assay (ELISA) kits (Jiancheng, Nanjing, China), according to the manufacturer’s instructions.

### 2.6. Determination of Intestinal Permeability (DAO and D-Lac)

The activity of diamine oxidase (DAO, A088-2-1) was determined according to the instructions of assay kits (Jiancheng, Nanjing, China). The level of D-lactose (D-Lac, H263) was determined using ELISA kits (Jiancheng, Nanjing, China), according to the manufacturer’s instructions.

### 2.7. Determination of Liver Function (ALT and AST)

The activities of alanine aminotransferase (ALT, C009-1-1) and aspartate aminotransferase (AST, C010-1-1) were determined according to the instructions of assay kits (Jiancheng, Nanjing, China). The activity of alkaline phosphatase (AKP, P0321M) was determined according to the instructions of assay kits (Beyotime Tech, Shanghai, China). 

### 2.8. Western Blotting

The tissues were lysed using RIPA lysis buffer (Beyotime Tech, Shanghai, China) with 1% PMSF (Beyotime Tech, Shanghai, China) and phosphatase inhibitor cocktail (BosterBio, Nanjing, China) to extract total protein. The concentration of proteins was determined by using a BCA assay (KeyGen Biotech, Nanjing, China). Tissue protein (30 μg/lane) was electrophoresed through SDS-PAGE at 90 V for 15 min and 120 V for 1 h, and then, electronically transferred to PVDF membranes at 260 mA for 1 h. After blocking with 5% de-fat milk for 2 h, the blots were incubated at 4 °C overnight with primary antibodies: β-actin (13E5) (purchased from Cell Signaling Technology (Boston, USA); ZO-1(21773-1-AP), occludin (27260-1-Ap), caspase-3 (66470-2-Ig), and JAK1 (66466-1-Ig) (purchased from Proteintech (Wuhan, China)); NF-κB p65 (ab32536), STAT3 (ab68153), and Nrf2 (ab76036) (purchased from Abcam (ab205718 and ab205719, Cambridge, UK)). After washing with TBST buffer three times, the HRP-conjugated secondary antibodies (Abcam, Cambridge, UK) were applied for 2 h at room temperature. Washing was performed again, and then the chemiluminescence signals were detected by using an ECL system (Biosharp, Hefei, China) and visualized by using a Chemiluminescence imager (Bio-Rad, California, USA). Finally, the images were analyzed using the Image Lab software. All expression levels were normalized to β-actin.

### 2.9. 16S rRNA-Based Microbiota Analysis

Novogene Technology Co., Ltd. (Beijing, China) was entrusted with the task of performing the 16S rRNA amplicon sequencing to analyze the fecal microbiomes for cecum samples collected from mice in the control group, the diquat model group, and the PAE-100 group.

#### 2.9.1. DNA Extraction and 16S rRNA Gene Sequencing

The genomic DNA of the samples was extracted using the cetyltrimethylammonium bromide (CTAB)/SDS method. The sequenced region 16S V3-V4 was selected for PCR amplification. A TruSeq® DNA PCR-Free Sample Preparation Kit was used for library construction. The constructed library was quantified by Qubit and Q-PCR. After the library was qualified, Illumina NovaSeq6000 was used for on-machine sequencing.

#### 2.9.2. Microbiota Analysis

The Illumina MiSeq sequencing and general data analysis were performed by a commercial company (Novogene, Beijing, China). First, the off-machine data (raw PE) obtained by Illumina NovaSeq sequencing were spliced and quality controlled to obtain Clean Tags, and then chimera filtering was performed to obtain effective data (Effective Tags) that could be used for subsequent analysis. The Uparse algorithm (Uparse v7.0.1001) was used to cluster the Effective Tags of all samples, and the sequences were clustered into operational taxonomic units (OTUs) with 97% identity (Identity), and then species annotation was performed on the sequences of OTUs. The top 10 species with the largest abundance at each taxonomic level (phylum, genus, and species) were selected for each sample or group, and a column accumulation chart of the relative abundance of species was generated. An alpha-diversity analysis and a beta-diversity analysis were performed using the Qiime software (Version 1.9.1). Then, R software through *T*-test, Wilcoxon rank-sum test, and Tukey test were used to analyze the species diversity between groups and whether or not the gender mean difference was significant. The LEfSe analysis was performed using the LEfSe software, and the filter value of LDA Score was set to 4. Species with significant differences between groups were analyzed by using R software to do a between-group *T*-test, and were plotted.

### 2.10. Statistical Analysis

All data are reported as mean ± standard deviation (SD). Means were compared by using one-way analysis of variance (ANOVA) followed by an LSD post hoc test; *p* < 0.05 was considered to be statistically significant. All experiments were performed at least 3 times. All statistical analyses were performed using the SPSS 26.0 software and plotted with the Graphpad Prism 9.0 software.

## 3. Results

### 3.1. Effects of PAE and Diquat on the Growth of Mice 

**Figure 2:**
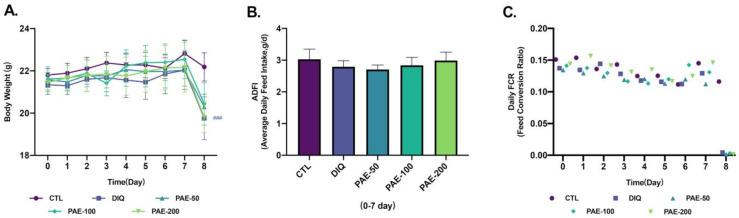
Effects of PAE on growth in diquat-induced mice: (**A**) Changes in body weight of mice (*n* = 8); (**B**) effects of PAE on average daily feed intake (ADFI) in diquat-treated mice (*n* = 8); (**C**) effects of PAE on daily feed conversion ratio (FCR) in diquat-treated mice (*n* = 8). ### *p* < 0.001 as compared with the CTL group.

As shown in Figure 2, oral gavage of PAE did not affect the body weight, average daily feed intake (ADFI), and daily feed conversion ratio (FCR) of mice before being challenged with diquat. After 24 h of intraperitoneal injection of diquat, the mice stopped feeding and showed significant weight loss as compared with the control group injected with normal saline.

### 3.2. PAE Helps Maintain Intestinal Barrier and Liver Function in Mice

**Figure 3:**
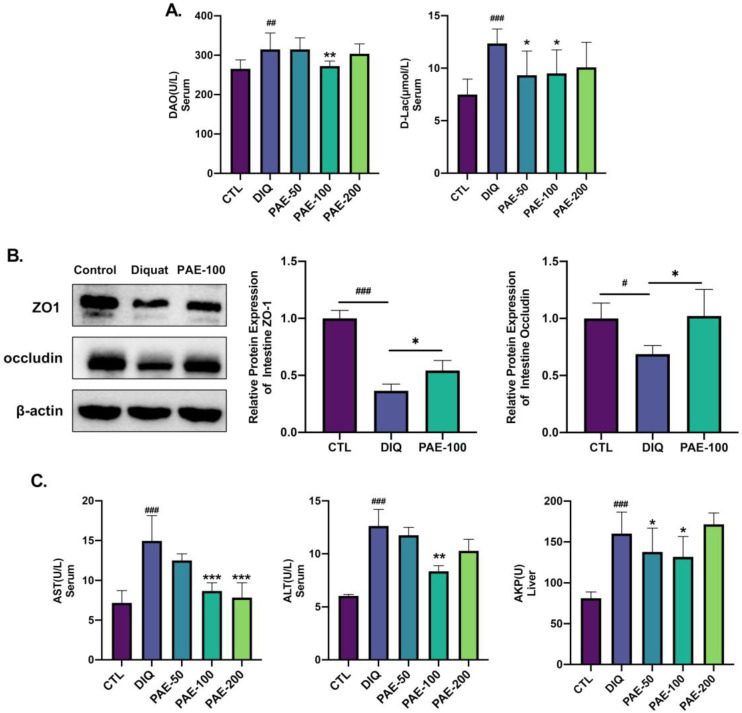
Effects of PAE on intestinal barrier and liver function in diquat-induced mice: (**A**) Analysis of intestinal permeability parameters (DAO, D-Lac) in serum by ELISA (*n* = 8); (**B**) analysis of the relative protein expression level (ZO-1, occludin) in intestine by WB (*n* = 3); (**C**) analysis of parameters of liver injuries (AST, ALT in serum, and AKP in liver) (*n* = 8). All data were represented as means ± SD. # *p* < 0.05, ## *p* < 0.01, and ### *p* < 0.001 as compared with the CTL group; * *p* < 0.05, ** *p* < 0.01, and *** *p* < 0.001 as compared with DIQ group.

Serum DAO and D-Lac levels positively correlated with intestinal permeability. Compared with the diquat-induced oxidative stress group, PAE-100 significantly inhibited the elevation of serum DAO level, while PAE-50/100 inhibited an increase in serum D-Lac content (Figure 3A). To further evaluate the intestinal barrier function, the expressions of tight junction proteins in the intestine of mice were quantified by Western blotting. Diquat resulted in significant downregulation of the protein expression levels of ZO-1 and occludin, but PAE-100 pretreatment effectively inhibited this downregulation (Figure 3B). The serum AST and ALT levels were used as biochemical parameters of liver injury. As shown in Figure 3C, PAE-100 pretreatment effectively inhibited the elevation of serum AST and ALT levels caused by diquat, and PAE-200 also effectively prevented the elevation of the serum AST level. In addition, PAE-50/100 attenuated diquat-induced elevation of the hepatic AKP level. 

### 3.3. PAE Enhances the Antioxidant Ability of the Intestine and Liver in Diquat-Treated Mice

**Figure 4:**
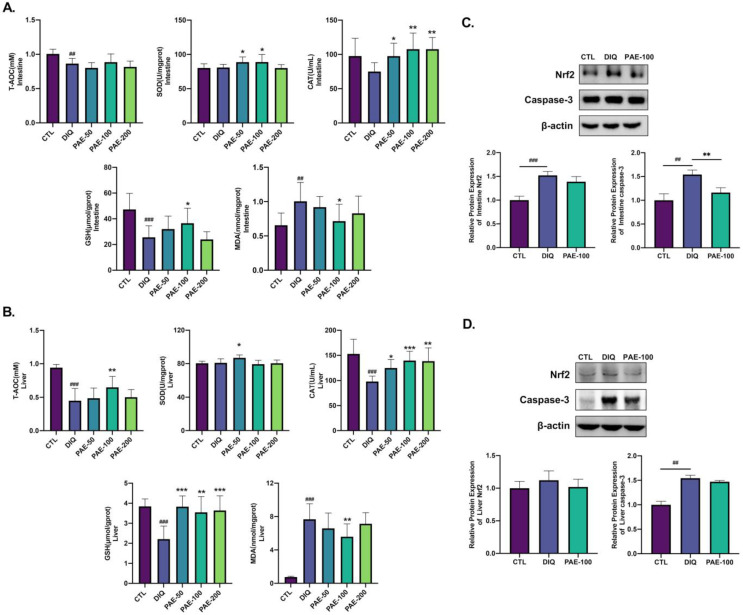
Effect of PAE on the antioxidant ability of the intestine and liver in diquat-treated mice: (**A**) Analysis of oxidative stress parameters (T-AOC, SOD, CAT, GSH, and MDA) in the intestine (*n* = 8); (**B**) analysis of oxidative stress parameters (T-AOC, SOD, CAT, GSH, and MDA) in the liver (*n* = 8); (**C**) analysis of the relative protein expression level (Nrf2 and caspase-3) in the intestine (*n* = 3); (**D**) analysis of the relative protein expression level (Nrf2 and caspase-3) in liver (*n* = 3). All data were represented as means ± SD. ## *p* < 0.01, and ### *p* < 0.001 as compared with control group; * *p* < 0.05, ** *p* < 0.01, and *** *p* < 0.001 as compared with the diquat group.

As shown in Figure 4A,B, it is obvious that PAE-50 increased the activity of SOD in the intestine and liver and PAE-100 increased the intestinal SOD activity, but diquat had no effect on SOD. Pretreatment with PAE significantly inhibited the diquat-induced activity and decreased CAT both in the intestine and liver. Similarly, diquat significantly decreased the levels of intestinal and hepatic GSH as compared with the control group; however, PAE pretreatment mitigated this decline in GSH levels in the liver, and only PAE-100 had a decent resistance to diquat in the intestine. PAE-100 effectively inhibited the production of lipid peroxidation product (MDA) induced by diquat in the intestine and liver. As shown in Figure 4C,D, diquat increased the protein expression levels of Nrf2 in the intestine and caspase-3 both in the intestine and liver. PAE pretreatment had no significant effect on the expression of Nrf2, but reduced the activation of caspase-3 caused by diquat.

### 3.4. Effects of PAE on Diquat-Induced Inflammation of the Intestine and Liver in Mice 

**Figure 5:**
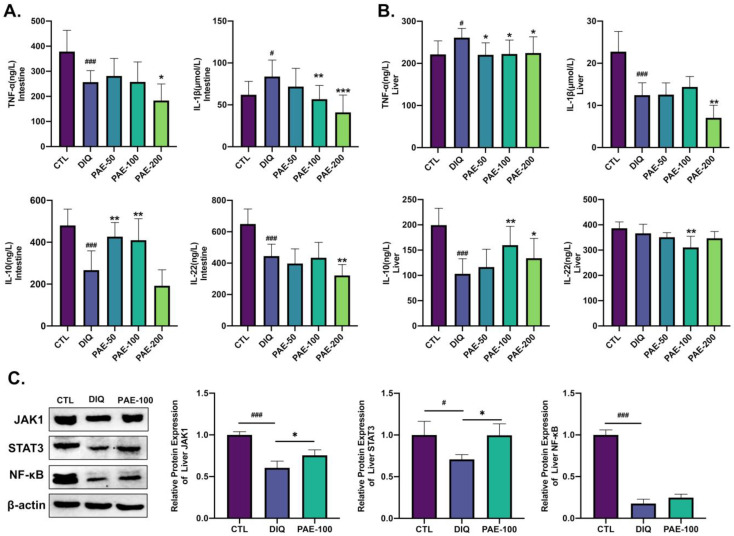
Effect of PAE on inflammation of the intestine and liver in diquat-treated mice: (**A**) Analysis of proinflammatory cytokines (TNF-α and IL-1β) and anti-inflammatory cytokines (IL-10 and IL-22) in the intestine (*n* = 8); (**B**) analysis of proinflammatory cytokines (TNF-α and IL-1β) and anti-inflammatory cytokines (IL-10 and IL-22) in the liver (*n* = 8); (**C**) analysis of the relative protein expression levels (JAK1, STAT3, and NF-κB) in the liver (*n* = 3). All data were represented as means ± SD. # *p* < 0.05, and ### *p* < 0.001 as compared with the control group; * *p* < 0.05, ** *p* < 0.01, and *** *p* < 0.001 as compared with the diquat group.

As shown in Figure 5, diquat decreased the level of TNF-α but increased the level of IL-1β in the intestine (Figure 5A). PAE-200 inhibited the levels of both TNF-α and IL-1β, and PAE-100 also had a significant inhibitory effect on IL-1β in the intestine. In contrast, diquat increased the level of TNF-α in the liver, while PAE pretreatment inhibited increased hepatic TNF-α. Meanwhile, diquat reduced the level of hepatic IL-1β, and PAE-200 further enhanced this decrease (Figure 5B). Diquat reduced the level of IL-10 both in the intestine and liver. PAE-50/100 significantly inhibited the reduction of intestinal IL-10 level, and PAE-100/200 significantly inhibited the reduction of hepatic IL-10 level induced by diquat in the liver (Figure 5A,B). Diquat significantly reduced the level of IL-22 in the intestine, but not in the liver, PAE-100 and PAE-200, respectively, promoted this reduction in the intestine and liver (Figure 5A,B). Then, we detected the expression levels of IL-10-mediated Janus tyrosine kinase 1 (JAK1)/signal transducer and activator of transcription 3 (STAT3) pathway protein and the classical inflammatory pathway protein nuclear factor kappa B (NF-κB) in the liver. As shown in Figure 5C, diquat significantly reduced the protein expression levels of JAK1, STAT3, and NF-κB. PAE-100 significantly inhibited the diquat-induced decrease of the JAK1 and STAT3 protein expression levels, but had no significant effect on the NF-κB level.

### 3.5. Effects of PAE on Gut Microbes in the Diquat-Treated Mice

#### 3.5.1. Effects of PAE on Gut Microbial Community in Diquat-Treated Mice

**Figure 6:**
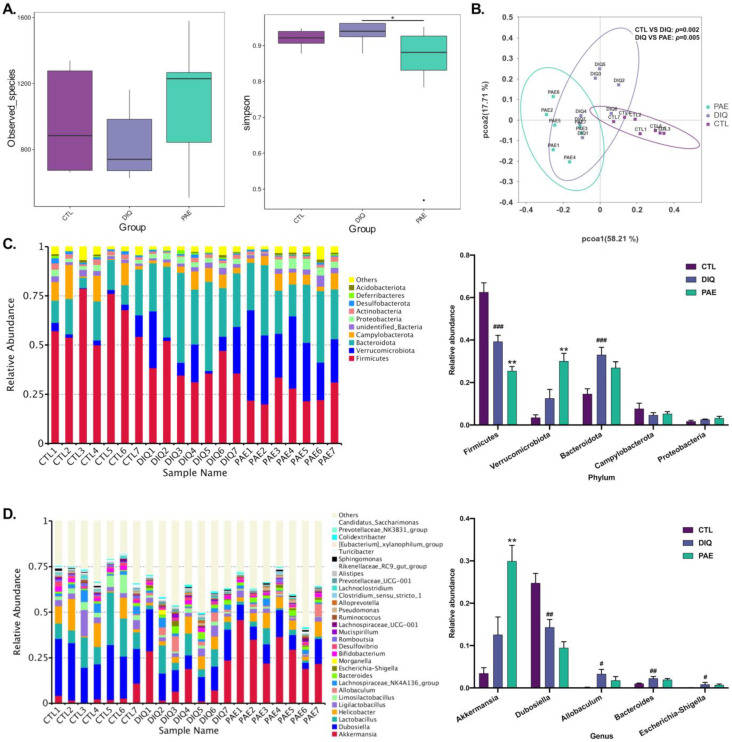
Effects of PAE on microbial community of cecum in the diquat-treated mice: (**A**) Alpha-diversity analysis (Observed_species and Simpson’s index); (**B**) beta-diversity analysis based on weighted Unifrac (PCoA); (**C**) relative abundance of the top 10 intestinal microbiota at the phylum level; (**D**) relative abundance of the top 30 intestinal microbiota at the genus level. PAE, PAE-100 (100 mg/kg) group. All data were represented as means ± SD, *n* = 7. # *p* < 0.05, ## *p* < 0.01, and ### *p* < 0.001 as compared with the control group; * *p* < 0.05 and ** *p* < 0.01 as compared with the diquat-treated group.

The results of the alpha-diversity analysis are shown in Figure 6A. Diquat downregulated the observed species in mice, but was not considered to be statistically significant, while PAE pretreatment lessened this result. A higher Simpson index was observed in the diquat-treated group, whereas PAE significantly reduced the Simpson index. The beta-diversity analysis is shown in the principal coordinates analysis (PCoA) scatterplot (Figure 6B), the data of the weighted UniFrac PCoA analyzed by Adonis illustrated that the difference between the control or PAE-pretreated groups and the diquat-treated group was significant.

Figure 6C shows the top 10 microbial phyla in the cecum of the mice. Changes in the relative abundance of microbes at the phylum indicated the effects of PAE and diquat on gut microbiota structure. After treatment, Firmicutes (62.6, 39.3, 25.5%), Verrucomicrobia (3.5, 12.6, 30%), Bacteroidetes (14.7, 33, and 27%), Campylobacterota (7.7, 4.7, and 5.3%), and Proteobacteria (1.8, 2.6, and 3.3%) were the top phyla in the control, the diquat-treated, and the PAE-100 pretreatment groups. Apparently, PAE pretreatment greatly increased the relative abundance of Verrucomicrobia. We calculated the abundance ratio of Bacteroidetes/Firmicutes; the diquat-treated group (84.1%) had a much higher ratio than the control (23.5%), and PAE significantly exacerbated this change (105.9%). The analysis of the relative abundance of microbes at the genus level is shown in Figure 6D. There were significant changes in the gut microbial community in mice treated with PAE and diquat. Diquat significantly decreased the relative abundance of *Dubosialla,* while increasing the relative abundance of the harmful flora (*Allobaculum*, *Bacteroides*, and *Escherichia-Shigella*). PAE greatly increased the relative abundance of gut *Akkermansia*, and was able to slightly reduce the diquat-induced increase in the abundance of *Allobaculum* and *Escherichia-Shigella*.

#### 3.5.2. Effects of PAE on Taxonomic Biomarkers of Gut Microbiota in the Diquat-Treated Mice

**Figure 7:**
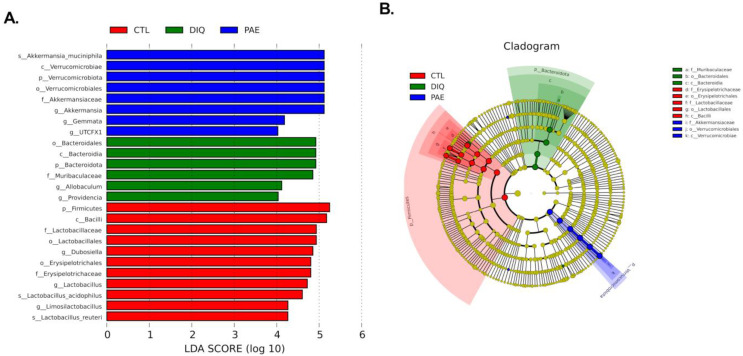
The analysis of LEfSe and LDA based on OTUs characterized the microbiomes of cecum in mice: (**A**) LDA score histogram shows taxonomic biomarkers (the LDA score (log10) > 4); (**B**) LEfSe evolutionary cladogram shows the phylogenetic distribution of the cecal microbe. PAE, PAE-100 (100 mg/kg) group.

By utilizing a linear discriminant analysis (LDA) effect size (LEfSe), the cecal microbial differential species were evaluated to identify the specific bacteria in the diquat and PAE groups. Figure 7A shows the species with significant differences, represented by the LDA score > 4.0, which reflects the degree of influence of species with significant differences between groups. At the phylum level, diquat significantly increased the abundance of Bacteroidota, while PAE pretreatment significantly increased the abundance of Verrucomicrobiota. At the genus level, diquat significantly increased the abundance of *Allobaculum* and *Muribaculaceae*, while PAE pretreatment significantly increased the abundance of Akkermansia, *Gemmata*, and *UTCFX1*. At the species level, *Akkermansia muciniphila* in the PAE pretreatment group was significantly different from the other groups.

## 4. Discussion

In recent years, the therapeutic potential of PAE has been increasingly exploited. Our study suggests that PAE can significantly alleviate oxidative stress in diquat-treated mice, while showing a certain inhibitory effect on oxidative stress-induced apoptosis (the activation of caspase-3). PAE pretreatment can significantly reduce the lipid peroxidation product (MDA) and can improve the antioxidant capacity (T-AOC) by increasing the activity of antioxidant enzymes (SOD and CAT) and promoting the production of antioxidants (GSH). In addition, PAE-100 (100 mg/kg) had the best antioxidative stress effect as compared with PAE-50 (50 mg/kg) and PAE-200 (200 mg/kg) in diquat-treated mice. This phenomenon may be explained by a “hormetic dose–response model”. Hormesis is a biphasic dose–response model that typically displays a “J-shaped” (or inverted “U-shaped”) curve, depending on the endpoints being drawn, with both stimulatory and inhibitory phases [28]. Hormetic dose–responses are common to the traditional Chinese medicine field, and many forms of preconditioning are likely to act through hormesis-based mechanisms [28,29]. When the intestinal barrier is disrupted, the transcellular electrical resistance decreases and the paracellular permeability for tracers increases, such as diamine oxidase (DAO), D-lactate (D-Lac), endotoxin (ET), and translocating bacteria [30]. In our study, PAE was shown to attenuate a decrease in the expression of TJ proteins (ZO-1 and occludin) in the small intestine of mice tissue and to maintain the intestinal permeability by reducing the extravasation of DAO and D-Lac from the gut into the blood. Other studies have also demonstrated the repairability of PAE on ulcerative colitis (UC) mice by improving the TJs and upregulating their associated proteins in the inflamed colon tissues [31,32,33]. A few of the enzymes contained in hepatocytes can be measured in the serum and used to assess liver function, which can be divided into two categories: enzymes that mainly reflect cholestasis, such as the alkaline phosphatase (AKP), and those that primarily reflect hepatocellular necrosis, such as the alanine aminotransferase (ALT) and aspartate aminotransferase (AST) [34]. PAE can maintain liver function and reduce liver damage by reducing the generation of AKP as well as reducing AST and ALT levels in the blood. This suggests that PAE may act as a potential hepatoprotectant against liver damage.

Nuclear factor kappa B (NF-κB) has been considered to be a classic proinflammatory signal, mainly due to the activation of NF-κB by proinflammatory cytokines such as IL-1β and TNF-α, and the role of NF-κB in the expression of other proinflammatory genes including cytokines, chemokines, and adhesion molecules [35]. In our study, we found that hepatic NF-κB expression was significantly decreased after diquat treatment, which was consistent with the IL-1β results. PAE had no strong effect on the NF-κB signaling pathway, but could significantly upregulate the levels of IL-10 both in the intestine and the liver and activate the JAK1/STAT3 signaling pathway. A similar finding in other research showed that PAE promoted wound healing by enhancing the activation of JAK1/2 and STAT3, but this was irrespective of NF-κB and Wnt signaling [36]. However, on ethanol-induced gastric ulcer mice, PAE was capable of lowering the stimulation of the nucleotide-binding domain-like receptor family pyrin domain-covering 3 (NLRP3)/caspase-1 channel, while impacting inflammation by downregulating proinflammatory cytokines (IL-18, TNF-α, IL-6, and IL-1β) [24]. Instead of decreasing the proinflammatory capacity, we believe that PAE primarily mitigates diquat-triggered inflammation by improving the anti-inflammatory capacity, particularly IL-10 and its mediated JAK1/STAT3 signaling pathway. This difference may be related to the immune properties of the liver, which default immune status is anti-inflammatory or immunotolerant [14], or could be due to inconsistencies in inducing drugs and models. This result needs further study.

A growing number of studies have recently shown that intestinal functions and gut microbiota are closely related, which may be jointly explained by the “leaky gut hypothesis” and the “dysbiosis hypothesis” [10,11,37]. For instance, intestinal dysbiosis and endotoxemia greatly affect the cirrhotics in relation to major complications and prognosis [37]. In this study, we found that diquat, indeed, caused intestinal flora imbalance by increasing the abundance of Bacteroidetes and Proteobactoria and decreasing Firmicutes, while causing leaky gut, which was manifested by gut bacterial metabolism leak of product (D-Lac). PAE pretreatment could improve alpha species diversity decline caused by diquat, while increasing the abundance of Verrucomicrobia and decreasing the abundance of Bacteroidetes. It has been reported that PAE increased the amounts of probiotics such as *Lactobacillus* and regulated the structure of the flora to alleviate dextran sulfate sodium (DSS)-induced UC in rats [33]. We have demonstrated a novel finding that PAE pretreatment can greatly increase the abundance of Verrucomicrobiota, *Akkermansia,* and *Akkermansia muciniphila* (*A. muciniphila*) in diquat-treated mice. The abundance of *A. muciniphila* has been increased after fasting in hamsters [38]. Therefore, we think that the improved abundance of *A. muciniphila* in the gut of diquat-treated mice, unlike the dominant species in the PAE group, may be a result of the poor health of the mice and their inability to eat after injection of diquat.

*A. muciniphila*, an intestinal symbiont that colonizes in the mucosal layer and uses mucin as energy source, is considered to be a promising next-generation probiotic [39,40]. According to numerous studies on the gut microbiota, *A. muciniphila* has been linked to a variety of metabolic diseases, including overweight, obesity, type 2 diabetes mellitus (T2DM), as well as particular gastrointestinal disorders, neurodegenerative conditions, and even cancers [41]. The potential pathogenicity of *A. muciniphila* is mainly due to the fact that degrading mucin itself is a pathogen-like behavior [42,43]. However, mucin degradation is considered to be a normal process in the intestinal self-renewal balance [44]. As a mucin degrader, *A. muciniphila* primarily colonizes the outer mucus layer, as opposed to the inner zone which still has a final physical barrier, such as the colonization of pathogens [45]. It has been reported that even when the abundance of *A. muciniphila* reached a high level of 60% in humans following broad-spectrum antibiotic treatment, no significant gastrointestinal disorders occurred [46]. At present, there is no report on any relevant disease or sign of pathogenicity of *Akkermansia* [44]. Multiple studies have indicated that restoring *A. muciniphila* levels in mice reduced the severity of alcoholic liver disease, colitis-associated carcinogenesis, and inflammatory bowel disease [47,48,49,50,51]. *A. muciniphila* participates in the host immune regulation, and also enhances the integrity of the intestinal epithelial cells, thereby, fortifying the impaired gut barrier [52]. *A. muciniphila* exposure has been shown to increase the expression of junctional integrity markers such as integrin-β1, E-cadherin, ZO-1, and occludin in cells [53]. *A. muciniphila* has been shown to influence the immune cell composition in mesenteric lymph nodes, shown by an increase in total B cell population and a decrease in total T cell and neutrophil populations [54]. Studies have demonstrated that *A. muciniphila* highly correlated with IL-10 in vivo and in vitro; both live and pasteurized *A. muciniphila* could restore the downregulation of IL-10 in UC mice and in H7N9-infected mice [55,56]. In vitro, *A. muciniphila* could increase IL-10 release in whole blood ex vivo LPS-stimulated mice as well as *Porphyromonas gingivalis*-infected bone marrow macrophages (BMMϕ) [54,57]. Studies have shown that different probiotic strains could exert their antioxidant capacity in different ways. Probiotics can regulate the redox status of organisms through their metal ion chelation capacity, antioxidant system and signaling pathways, composition of gut microbiota, and metabolism of short-chain fatty acids (SCFAs) [58,59]. In recent years, SCFAs have attracted considerable attention. Carbohydrate fermentation by anaerobes provides the host with important SCFAs such as acetate, propionate, and butyrate [60], which are normally responsible for maintaining microbial homeostasis [61,62]. *A. muciniphila*, a beneficial anaerobic bacteria, can potentially provide various health benefits through increasing SCFAs production [63]. Oral epigallocatechin-3-gallate (EGCG) can relieve DSS-induced colitis and oxidative stress, which is closely associated with SCFAs-producing bacteria *Akkermansia* and SCFAs, as further evidenced by prophylaxis and fecal microbiota transplantation (FMT) [64]. Metformin has been suggested to also alter the composition of the gut microbiota by increasing *A. muciniphila* and several SCFA-producing microbes for the treatment of patients with type 2 diabetes [65]. Therefore, we speculate that the significantly increased abundance of *A. muciniphila* in PAE-pretreated mice is associated with intestinal barrier function, anti-inflammatory capacity, and SCFAs production against diquat-induced oxidative stress and inflammation. However, this requires further testing to validate the effectiveness of PAE in promoting intestinal *A. muciniphila* colonization and the relationship between *A. muciniphila* and antioxidant and anti-inflammatory. The results of this study may provide a direction for opening some future disease treatment strategies that PAE can effectively increase the abundance of *A. muciniphila* in the gut.

## 5. Conclusions

In this study, we found that *Periplaneta americana* extract pretreatment could effectively reduce diquat-induced oxidative stress in the intestine and liver, and could alleviate inflammation mainly by increasing anti-inflammatory ability, that is, activating the IL-10/JAK1/STAT3 signaling pathway. In addition, a novel finding of this study is that PAE can significantly increase the abundance of *Akkermansia muciniphila* in the gut, which may further help to reduce oxidative stress and inflammatory responses. 

## Data Availability

Data are contained within the article.
